# Preemptive intrathecal administration of endomorphins relieves inflammatory pain in male mice via inhibition of p38 MAPK signaling and regulation of inflammatory cytokines

**DOI:** 10.1186/s12974-018-1358-3

**Published:** 2018-11-15

**Authors:** Ting Zhang, Nan Zhang, Run Zhang, Weidong Zhao, Yong Chen, Zilong Wang, Biao Xu, Mengna Zhang, Xuerui Shi, Qinqin Zhang, Yuanyuan Guo, Jian Xiao, Dan Chen, Quan Fang

**Affiliations:** 1 Key Laboratory of Preclinical Study for New Drugs of Gansu Province, and Institute of Physiology, School of Basic Medical Sciences, Lanzhou University, 199 Donggang West Road, Lanzhou, 730000 People’s Republic of China; 2 Department of Neurology, School of Medicine, Duke University, Durham, North Carolina 27710 USA

**Keywords:** Endomorphin, Preemptive analgesic, Inflammatory pain, p38 MAPK, Inflammatory cytokines

## Abstract

**Background:**

Preemptive administration of analgesic drugs reduces perceived pain and prolongs duration of antinociceptive action. Whereas several lines of evidence suggest that endomorphins, the endogenous mu-opioid agonists, attenuate acute and chronic pain at the spinal level, their preemptive analgesic effects remain to be determined. In this study, we evaluated the anti-allodynic activities of endomorphins and explored their mechanisms of action after preemptive administration in a mouse model of inflammatory pain.

**Methods:**

The anti-allodynic activities of preemptive intrathecal administration of endomorphin-1 and endomorphin-2 were investigated in complete Freund’s adjuvant (CFA)-induced inflammatory pain model and paw incision-induced postoperative pain model. The modulating effects of endomorphins on the expression of p38 mitogen-activated protein kinase (p38 MAPK) and inflammatory mediators in dorsal root ganglion (DRG) of CFA-treated mice were assayed by real-time reverse transcription-polymerase chain reaction (RT-PCR), Western blotting, or immunofluorescence staining.

**Results:**

Preemptive intrathecal injection of endomorphins dose-dependently attenuated CFA-induced mechanical allodynia via the mu-opioid receptor and significantly reversed paw incision-induced allodynia. In addition, CFA-caused increase of phosphorylated p38 MAPK in DRG was dramatically reduced by preemptive administration of endomorphins. Repeated intrathecal application of the specific p38 MAPK inhibitor SB203580 reduced CFA-induced mechanical allodynia as well. Further RT-PCR assay showed that endomorphins regulated the mRNA expression of inflammatory cytokines in DRGs induced by peripheral inflammation.

**Conclusions:**

Our findings reveal a novel mechanism by which preemptive treatment of endomorphins attenuates inflammatory pain through regulating the production of inflammatory cytokines in DRG neurons via inhibition of p38 MAPK phosphorylation.

**Electronic supplementary material:**

The online version of this article (10.1186/s12974-018-1358-3) contains supplementary material, which is available to authorized users.

## Background

Endomorphin-1 (EM-1) and endomorphin-2 (EM-2) were firstly isolated from bovine brain and exhibited high affinity and selectivity towards the mu-opioid receptor (MOR) [1]. It has been demonstrated that both EM-1 and EM-2 produced potent analgesic actions in inflammatory pain, which were reversed by the opioid receptor antagonist naloxone or β-funaltrexamine (β-FNA) [2–4]. For example, central administration of endomorphins alleviated formalin-induced nocifensive behaviors [4, 5]. Peripherally administered EM-1 significantly reduced the synovial vascular permeability in acute joint inflammation [6]. Moreover, Feehan et al. reported that the cyclized analogs of endomorphins produced potent and long-lasting analgesia in a rat model of inflammatory pain after intravenous or intrathecal administration [7]. In addition, histological studies elucidated that both EM-1 and EM-2 were expressed in macrophages and monocytes in lymph nodes during peripheral inflammation, suggesting the involvement of endomorphins in inflammatory pain [8].

Notably, compared with the opioid analgesic morphine, endomorphins induced more potent analgesia in neuropathic pain [9, 10] and had fewer side effects on reward, respiratory depression, and cardiovascular effects [11, 12]. However, central injection of endomorphins caused a short duration of antinociceptive action (less than 30 min) because of their poor enzymatic stability [13, 14]. It has been largely implicated that preemptive treatment with analgesics attenuates perceived pain for a prolonged duration, which might be explained by its ability to block the pathophysiological development of pain, including peripheral and central sensitizations. Briefly, peripheral sensitization is often accompanied by release of inflammatory mediators and decreases the threshold of terminal nerve endings, which leads to the enhancement of nociceptive pain [15]. Central sensitization results from an enhanced response which is provoked by hyperexcitability of the neurons in the dorsal horn of the spinal cord [16]. Both central and peripheral sensitizations are therefore the major causes of pain hypersensitivity. In theory, preemptive treatment with analgesics could block the noxious impulses and prevent central nervous system sensitization, contributing to more effective reduction of the nociceptive signals in a manner different to the treatment with analgesics after injury. Indeed, opioid analgesics, such as morphine and fentanyl, have well-demonstrated preemptive analgesic action in clinical and pre-clinical studies [17, 18]. To date, however, very little is known about the influence of preemptive administration of endomorphins on pain management.

Although the above evidences suggest endomorphins are potential pain therapeutics, their mechanisms await further elucidation. Immunohistochemical analysis revealed that the mu-opioid receptor was co-expressed with substance P (SP) and calcitonin gene-related peptide (CGRP) in DRG neurons [19, 20], and EM-1 diminished the release of SP and CGRP from primary afferent terminals [21], implying endomorphins may modulate stimulation-evoked release of SP and CGRP to alleviate pain. Accumulated evidence indicates that various inflammatory mediators including pro-inflammatory cytokines and chemokines in dorsal root ganglion (DRG) and spinal cord tissues are involved in the generation and maintenance of chronic pain [22–24]. In addition, extracellular pain stimuli aggravate the activation of mitogen-activated protein kinase (MAPK) pathways and transcription factors, which plays important roles in pain sensitization through regulating inflammatory mediators [25, 26]. Effects of endomorphins on inflammatory mediators have been documented in in vitro studies. Both EM-1 and EM-2 inhibited the release of inflammatory mediators, such as interleukin-12 (IL-12) and tumor necrosis factor-α (TNF-α) from macrophage cell line THP-1 [27, 28]. Opposite effects of endomorphins on the release of interleukin-1beta (IL-1β) have been observed, with a inhibitory effect reported in cultured rat peritoneal macrophages [29], but a stimulatory effect in macrophage cells line THP-1 [27]. Moreover, previous reports found that endomorphins can inhibit the phosphorylation of p38 MAPK induced by advanced glycation end products in cultured human umbilical vein endothelial cells, and similar results were also found in LPS-stimulated murine dendritic cells [30, 31]. These in vitro studies implicate that p38 MAPK and its possible downstream targets inflammatory mediators might contribute to the pain modulation of endomorphins.

In this study, we employed a chronic inflammatory pain model-induced by complete Freund’s adjuvant (CFA) to investigate the effects of preemptive intrathecal administration of EM-1 and EM-2 on the development of mechanical allodynia. We then determined the expression of the mu-opioid receptor in DRG neurons after inflammation and explored whether the MAPK signaling and inflammatory mediators in DRGs are downstream targets of preemptive analgesia of endomorphins. In addition, the preemptive analgesic effects of EM-1 and EM-2 were also evaluated at the spinal level in a postoperative pain model-induced by paw incision.

## Methods

### Drugs and reagents

EM-1 and EM-2 were prepared by manual solid-phase synthesis using standard *N*-fluorenylmethoxycarbonyl (Fmoc) chemistry as reported in our previous study [32]. Fmoc-protected amino acids (GL Biochem Ltd., China) were coupled with Rink amide 4-methybenzhydrylamine (MBHA) resin (Tianjin Nankai Hecheng Science & Technology Co., Ltd., China). The crude peptides were purified by preparative reversed-phase HPLC (RP-HPLC) and determined by electrospray ionization mass spectrometer (ESI-Q-TOF maXis-4G, Bruker Daltonics).

Naloxone, β-FNA, nor-binaltorphimine (nor-BNI), and naltrindole (NTI) were obtained from Sigma-Aldrich. The selective p38 MAPK inhibitor SB203580 was purchased from Beyotime Institute of Biotechnology and dissolved in 1% DMSO in saline. All other drugs were dissolved in sterilized saline and stored at − 20 °C.

### Experimental animals

Male Kunming mice (23 ± 2 g) were purchased from the Experimental Animal Center of Lanzhou University. Mice were housed in 12-h light-dark cycle and climate-controlled rooms at 22–24 °C with water and foods available. All behavioral tests were performed between 8:00 am and 6:00 pm, and all animals were used only once. Best efforts were made to minimize numbers of animals used and their suffering. The animal experimentation protocols were in compliance with the ARRIVE guidelines [33] and approved by the Ethics Committee of Lanzhou University and carried out in accordance with the European Community guidelines for the use of experimental animals (2010/63/EU).

### Drug administration

Intrathecal (i.t.) injection procedure was performed as previously described [34]. Briefly, a 25-μl microsyringe was inserted between L5 and L6 segment, and the flick or formation of an ‘S’ shape by the tail was considered as a successful injection. Drugs were injected into subarachnoid space with a total volume of 5 μl at a constant rate of 10 μl/min.

The opioid antagonists naloxone, β-FNA, nor-BNI, and NTI were injected 10 min, 4 h, 30 min, and 10 min prior to the administration of endomorphins, respectively. The doses and time of administration of these antagonists were chosen on the basis of our previous report [32].

### Inflammatory pain model and behavior test

CFA inflammatory pain was conducted following previous protocol [35]. Briefly, intraplantar (i.pl.) injection of 20 μl CFA (1 mg/ml, Sigma Aldrich, USA) was performed to induce chronic inflammation. In order to evaluate the effects of preemptive administration of endomorphins, animals received an intrathecal injection of saline or endomorphins (7.5, 15, and 30 nmol) 10 min prior to CFA administration. The force (g) at which mice withdrew the hindpaw was recorded automatically using electronic von Frey apparatus (Ugo Basile, Italy), and a cutoff value of 10 g was used to prevent tissue damage. The baseline withdrawal threshold of naïve mice was measured prior to drugs administration, and mechanical allodynia was measured at 4 h and daily for 7 days after CFA injection. To evaluate the role of p38 MAPK signal pathway in CFA-induced inflammatory pain, mice were received intrathecal injection of saline or SB203580 once a day, and mechanical allodynia was measured at 30-min and 90-min post-injection. Each time point was measured three times at 2-min intervals. The raw data from CFA-induced inflammatory pain assays were converted to area under the curve (AUC). The AUC depicting total paw withdrawal threshold versus time was computed by trapezoidal approximation. Upon termination of the experiment, all animals were subjected to euthanasia using CO_2_.

### Paw incision-induced post-operative pain model

We followed the procedure for the paw incision model as previously described [36–38]. Briefly, mice were anesthetized with isoflurane, and a 5-mm longitudinal incision was made though skin, fascia, and muscle on the plantar surface of the right hindpaw. The plantaris muscle was elevated and incised longitudinally. After gentle pressure for hemostasis, the skin was sutured and coated with antibiotic ointment. The effects of preemptive endomorphins were measured by von Frey test 2 h to 4 days after incision as indicated in Fig. 2.

### Protein extraction and Western blot analysis

For Western blot analysis, routine procedures were followed as previously described [39]. Briefly, ipsilateral L4-L6 DRG tissues were removed from CO_2_ euthanized mice, and homogenized in cell lysis buffer containing 0.1 M phenylmethylsulfonyl fluoride (PMSF). The protein concentration of each sample was estimated by BCA assay (Beijing Solarbio Science & Technology Co., Ltd., China). Equal content of proteins (80 μg) were separated by 12% SDS-polyacrylamide gelelectrophoresis and then transferred onto PVDF membrane (Bio-Rad Laboratories, China). A blocking step for 90 min at room temperature with 6% skim milk was followed by incubation with primary antibodies: rabbit anti-phosphorylated ERK1/2 antibody (P-ERK1/2) (1:1000, Cell Signaling Technology #9101, China), rabbit anti-ERK1/2 antibody (1:1000, Cell Signaling Technology #9102, China), mouse anti-p38 MAPK antibody (1:1000, Beyotime Biotechnology, AM065, China), or rabbit anti-phosphorylated p38 MAPK antibody (P-p38 MAPK) (1:1000, Cell Signaling Technology #4511, China) overnight at 4 °C. Afterwards, PVDF membranes were washed with TBST (50 mM Tris (pH 7.5), 100 mM NaCl, and 0.1% Tween 20) and incubated with secondary antibody (1:1000; HRP-labeled goat anti-mouse LgG (H + L), A0216 and HRP-labeled goat anti-rabbit LgG (H + L), A0208; Beyotime Institute of Biotechnology, China) for 2 h at room temperature. Protein signals were then visualized with enhanced chemiluminescence (Amersham Pharmacia Biotech, UK) and quantified by Image J software. The levels of P-p38 MAPK and P-ERK1/2 were normalized against corresponding total p38 MAPK and ERK1/2 level.

### Immunofluorescence staining

Immunofluorescence staining was performed following the previous report [39]. Mice were deeply anesthetized with pentobarbital sodium (100 mg/kg, intraperitoneal) and transcardially perfused with PBS followed by ice-cold 4% paraformaldehyde (PFA, Sigma-Aldrich, China). The ipsilateral L4 DRGs were dissected, post-fixed in 4% PFA, cryoprotected with 20% sucrose solutions, and then embedded in Tissue-Tek O.C.T. (Tissue-Tek, Sakura Finetek USA, CA). Immunohistochemistry staining was performed on 12-μm thickness sections. Briefly, sections were pre-incubated with 5% normal donkey serum to block the unspecific binding at room temperature for 45 min. Then, sections were incubated with the primary antibodies (Additional file 1: Table S1), including rabbit monoclonal P-p38 MAPK antibody or guinea pig monoclonal mu-opioid receptor antibody, overnight at 4 °C. This was followed by incubation with the corresponding secondary antibodies for 2 h at room temperature. For double immunofluorescence labeling, the two primary antibodies were applied simultaneously, followed by incubation with the corresponding secondary antibodies. Sections were examined using an Olympus fluorescence microscope equipped with high-resolution CCD Spot camera.

For each animal, four to six DRG sections were analyzed. Each group has at least four mice. The density threshold for the positive immunoreactivity (IR) was determined by averaging two or three cell bodies in each section that were judged to be minimally positive. All neurons for which the mean density exceeded the threshold were counted as positive, and the positive cells were expressed as a percentage of total DRG neurons.

Co-localization images were made into figures using Adobe Photoshop (Adobe Systems Incorporated, USA), and only minor adjustments to the contrast and brightness settings were applied where necessary. For evaluating co-localization of markers, six randomly selected DRG sections from each mouse were chosen for each pair of markers. Counts were made of the number of positive for each marker (P-p38 MAPK or the mu-opioid receptor), the number of the expression of both antigens and the total number of neurons. Result was presented as a percentage of total DRG neurons.

### RNA extraction, reverse transcription and real-time quantitative PCR

The L4-L6 ipsilateral DRGs were quickly removed from CO_2_ euthanized mice and homogenized in Trizol reagent (TaKaRa, China). Two micrograms of total RNA was reversed transcribed using a manufacturer’s protocol (TaKaRa, China). Real-time quantitative PCR reactions were performed using the SYBR Premix Ex Taq™II kit (TaKaRa, China) and an Agilent MX3005P (Agilent, USA) detection system. Primer sequences used for RT-PCR (Genbank, National Center for Biotechnology Information; www.ncbi.nlm.nih.gov) were displayed in Table 1. The PCR amplifications conditions were as follows: hold: 95 °C for 60 s; cycling: 95 °C for 5 s, 60 °C for 60 s, 40 cycles; and melt from 60 to 95 °C, and the relative expressions of genes were normalized to the internal reference GAPDH and analyzed using 2^− ΔΔCT^ method. In addition, the detection timing was chosen on the basis of the previous report [40].

**Table 1:** Primer sequences

Gene	Primer sequence(5′-3′)	GenBank accession no.
GAPDH	GGTTGTCTCCTGCGACTTCA (forward)	NM_001115114.1
	GGGTGGTCCAGGGTTTCTTA(reverse)	
IL-1β	ACTGGTACATCAGCACCTCAC (forward)	NM_008361.4
	TAGAAACAGTCCAGCCCATAC (reverse)	
IL-10	CACTACCAAAGCCACAAG (forward)	NM_010548.2
	GGAGTCGGTTAGCAGTATG (reverse)	
TNF-α	GAGAAGTTCCCAAATGGC (forward)	NM_013693.3
	ACTTGGTGGTTTGCTACG (reverse)	
CCL2	CAGCAAGATGATCCCAATG (forward)	NM_011333.3
	TGGTTCCGATCCAGGTTT (reverse)	
CCL3	ACTGACCTGGAACTGAATG (forward)	NM_011337.2
	GAAGAGTCCCTCGATGTG (reverse)	

### Statistical analysis

All data were presented as means ± S.E.M. The effective dose 50% of maximum response (ED_50_) values for antinociception 1 day after CFA were calculated using Graphpad Prism 5. For the time-course effects of endomorphins, data were analyzed using two-way ANOVA followed by Bonferroni post-hoc analysis, and corresponding AUC data were analyzed using one-way ANOVA followed by Dunnett or Bonferroni post-hoc test. For Western blot and immunohistochemistry assays, data were analyzed using two-tailed *t* test for the two groups’ comparison and one-way ANOVA for multi-group comparisons. For RT-PCR, data were analyzed using Mann-Whitney test. Probabilities of less than 5% (*P* < 0.05) were considered statistically significant.

## Results

### Preemptive intrathecal administration of EM-1 and EM-2 robustly reduced the development of mechanical allodynia

**Fig. 1:**
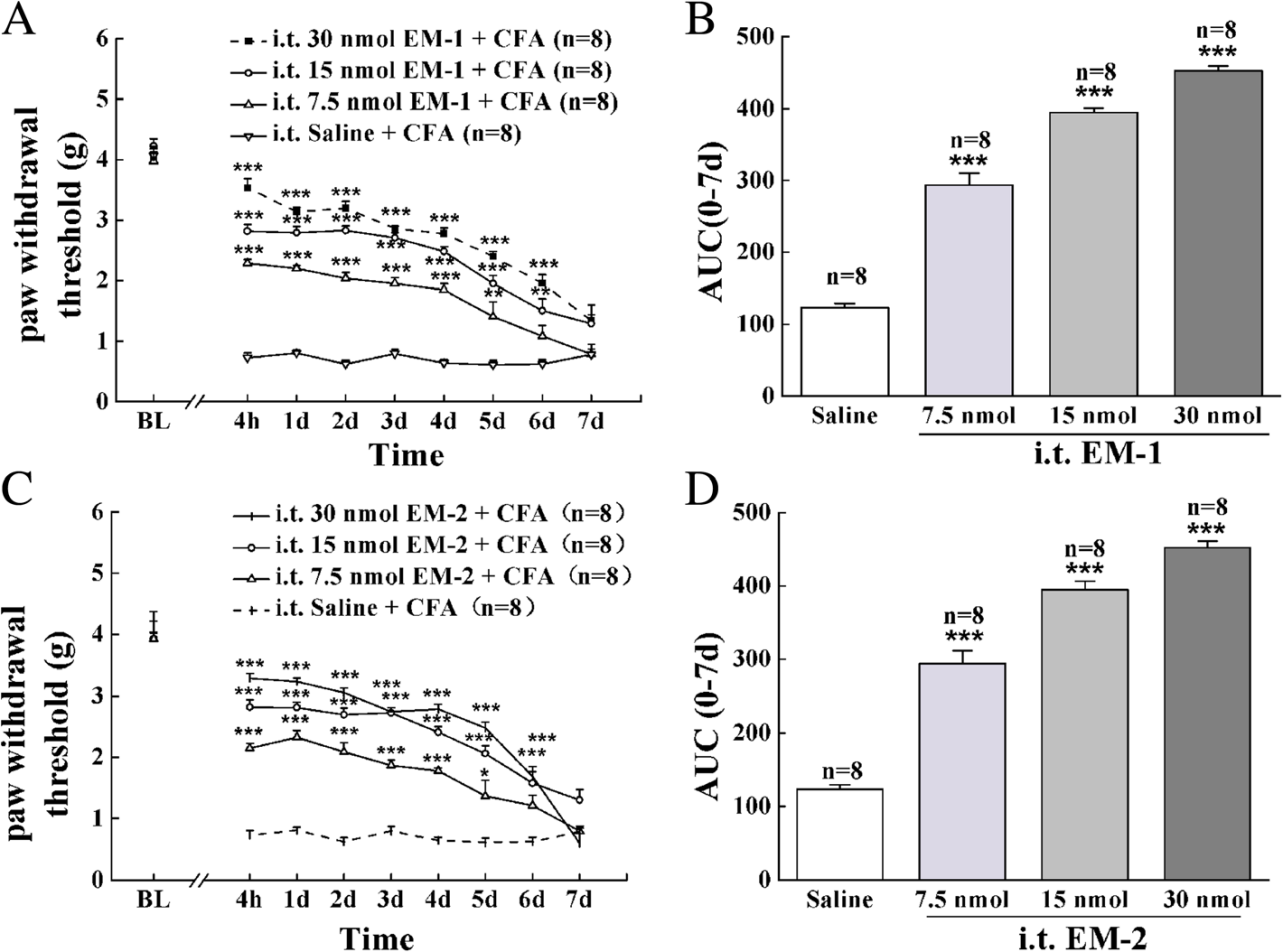
Preemptive intrathecal administration of EM-1 and EM-2 attenuated CFA-induced mechanical allodynia. Preemptive intrathecal administration of EM-1 (**a**, 7.5, 15, and 30 nmol) and EM-2 (**c**, 7.5, 15, and 30 nmol) dose- and time-dependently attenuated mechanical allodynia in male mice. Each value represents mean ± S.E.M. Group size is indicated in figures. ^*^P < 0.05, ^**^P < 0.01, and ^***^P < 0.001 indicate significant differences compared with the Saline + CFA group according to two-way ANOVA followed by Bonferroni post-hoc analysis. **b**, **d** AUC was calculated during 0–7 days from dose-response curve of EM-1 or EM-2, *P < 0.05, **P < 0.01, and ***P < 0.001 indicate significant differences compared with saline according to one-way ANOVA followed by Bonferroni post-hoc analysis

**Fig. 2:**
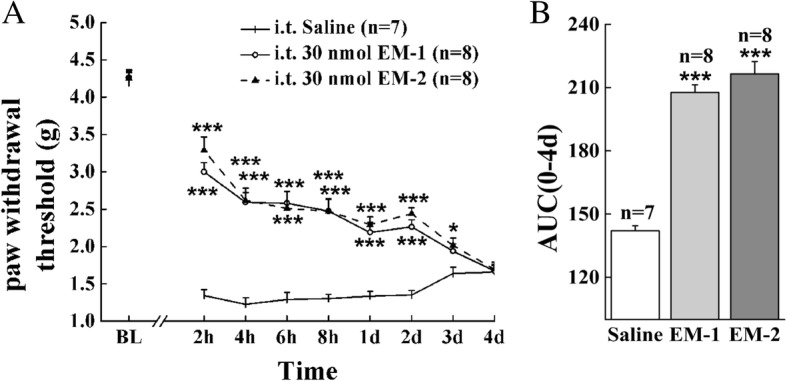
Preemptive administration of EM-1 and EM-2 robustly reduced the development of paw incision-induced mechanical allodynia. **a** Preemptive intrathecal administration of EM-1 (30 nmol, i.t.) and EM-2 (30 nmol, i.t.) 10 min before paw incision time-dependently attenuated mechanical allodynia in male mice. Each value represents mean ± S.E.M. Group size is indicated in figures. *P < 0.05 and ***P < 0.001 indicate significant differences compared with the Saline group according to two-way ANOVA followed by Bonferroni post-hoc analysis. **b** AUC was calculated during 0–4 days from time-response curve of EM-1 and EM-2, and ***P < 0.001 indicates significant differences compared with the Saline group according to one-way ANOVA followed by Bonferroni post-hoc analysis

Intraplantar injection of CFA induced a strong mechanical allodynia during the whole experimental period. As shown in Fig. 1a, c, both EM-1 and EM-2 dose-dependently attenuated mechanical sensitivity in male mice for 6 days when injected intrathecally 10 min before CFA treatment (*F*_24, 287_ = 11.4, *P* < 0.001; *F*_24, 287_ = 12.0, *P* < 0.001, respectively). The ED_50_ values for EM-1- and EM-2-induced preemptive antinociception 1 day after CFA were 10.11 (7.70–13.28) and 7.44 (0.68–81.80) nmol, respectively. Moreover, in paw incision-induced postoperative pain model, preemptive intrathecal administration of EM-1 (30 nmol) and EM-2 (30 nmol) also reversed the development of mechanical allodynia in male mice for 2 days (Fig. 2, *F*_16, 206_ = 8.12, *P* < 0.001).

### Effects of intrathecal administration of opioid antagonists on preemptive anti-allodynia induced by EM-1 and EM-2

**Fig. 3:**
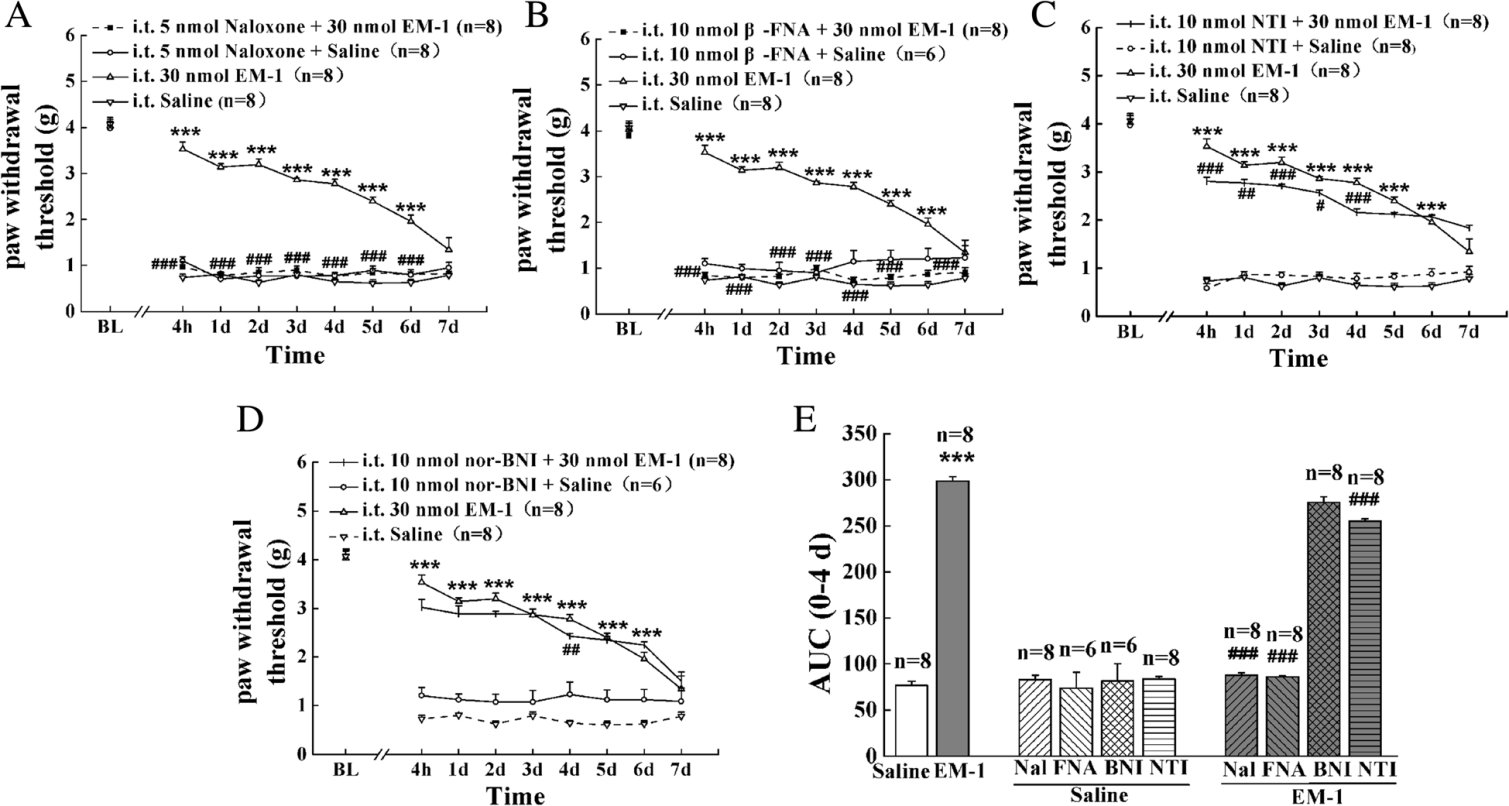
Effects of the opioid antagonists on the anti-allodynic activities of preemptive administration of EM-1. The opioid antagonists naloxone (5 nmol, i.t., (**a**)), β-FNA (10 nmol, i.t., (**b**)), NTI (10 nmol, i.t., (**c**)), and nor-BNI (10 nmol, i.t., (**d**)) were administered 10 min, 4 h, 10 min, and 30 min prior to EM-1 (30 nmol, i.t.) injection, respectively. Each value represents mean ± S.E.M. Group size is indicated in figures. *P < 0.05, **P < 0.01, and ***P < 0.001 indicate significant differences compared with the Saline group according to two-way ANOVA followed by Bonferroni post-hoc analysis, ^#^*P <* 0.05, ^##^*P <* 0.01, and ^###^*P <* 0.001 indicate significant differences compared with EM-1 group according to two-way ANOVA followed by Bonferroni post-hoc analysis. **e** AUC data were calculated during 0–4 days; *P < 0.05, **P < 0.01, and ***P < 0.001 indicate significant differences compared with the Saline group according to one-way ANOVA followed by Bonferroni post-hoc analysis. ^#^*P <* 0.05, ^##^*P* < 0.01, and ^###^*P <* 0.001 indicate significant differences compared with EM-1 group according to one-way ANOVA followed by Bonferroni post-hoc analysis

**Fig. 4:**
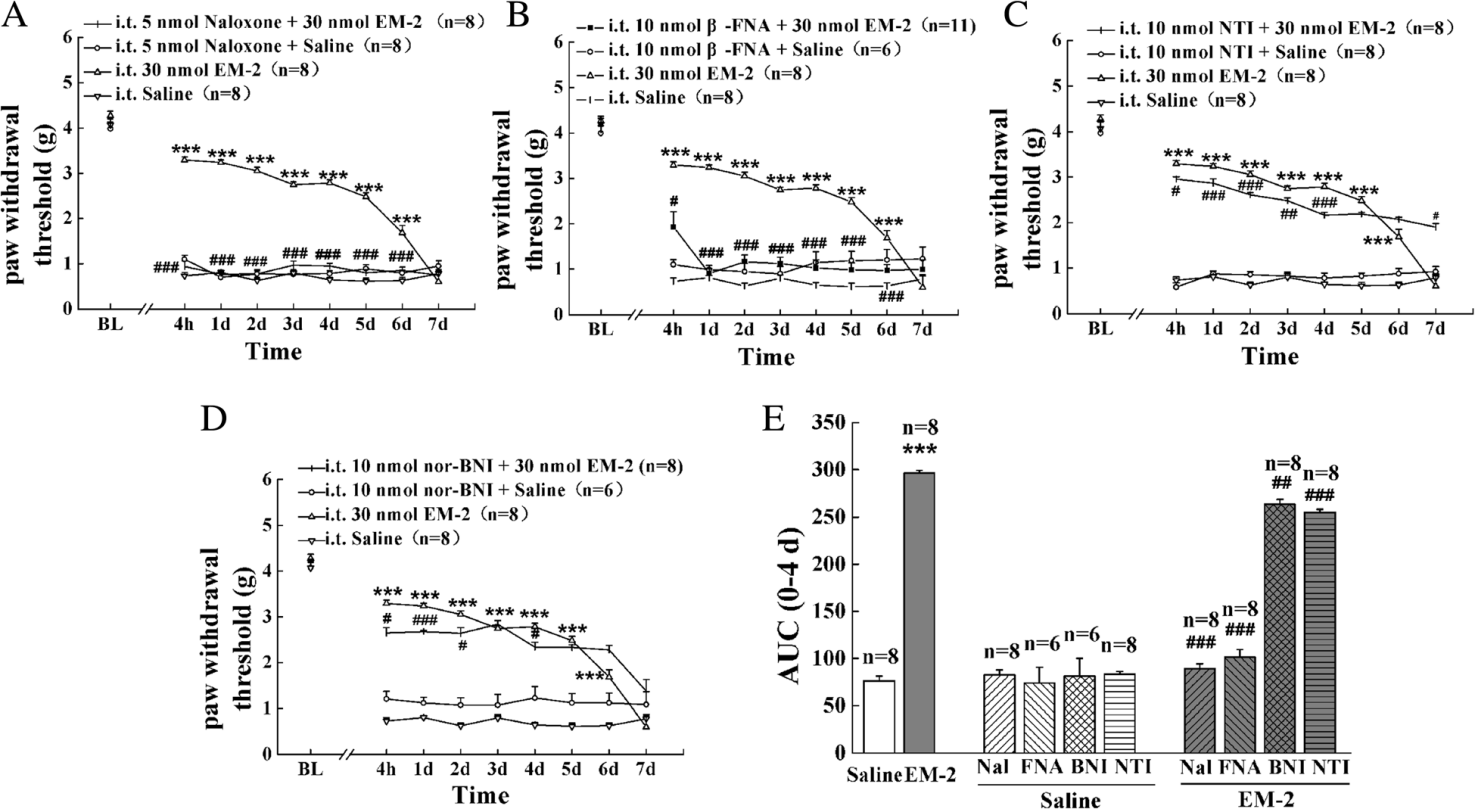
Effects of the opioid antagonists on the anti-allodynic activities of preemptive administration of EM-2. The opioid antagonists naloxone (5 nmol, i.t., (**a**)), β-FNA (10 nmol, i.t., (**b**)), NTI (10 nmol, i.t., (**c**)), and nor-BNI (10 nmol, i.t., (**d**)) were administered 10 min, 4 h, 10 min, and 30 min prior to EM-2 (30 nmol, i.t.) injection, respectively. Each value represents mean ± S.E.M. Group size is indicated in figures. *P < 0.05, **P < 0.01, and ***P < 0.001 indicate significant differences compared with the Saline group according to two-way ANOVA followed by Bonferroni post-hoc analysis; ^#^*P <* 0.05, ^##^*P <* 0.01, and ^###^*P <* 0.001 indicate significant differences compared with EM-2 group according to two-way ANOVA followed by Bonferroni post-hoc analysis. **e** AUC data were calculated during 0–4 days; *P < 0.05, **P < 0.01, and ***P < 0.001 indicate significant differences compared with Saline group according to one-way ANOVA followed by Bonferroni post-hoc analysis. ^#^*P <* 0.05, ^##^*P* < 0.01, and ^###^*P <* 0.001 indicate significant differences compared with the EM-2 group according to one-way ANOVA followed by Bonferroni post-hoc analysis

As shown in Figs. 3a and 4a, in CFA-induced inflammatory pain model, pretreatment with the nonselective opioid receptor antagonist naloxone (5 nmol, i.t.) completely reversed the anti-allodynic effects of EM-1 (30 nmol, i.t.) and EM-2 (30 nmol, i.t.) from 4 h to 4 days (Fig. 3a, *F*_24, 287_ = 18.7, *P* < 0.001; Fig. 4a, *F*_24, 287_ = 18.6, *P* < 0.001, respectively). In addition, the anti-allodynic effects of EM-1 and EM-2 from 4 h to 4 days were significantly blocked by the selective mu-opioid receptor antagonist β-FNA (10 nmol, i.t.) (Fig. 3b, *F*_24, 269_ = 15.5, *P* < 0.001; Fig. 4b, *F*_24, 269_ = 10.8, *P* < 0.001, respectively). Interestingly, the selective delta-opioid receptor antagonist NTI (10 nmol, i.t.) only partially inhibited the preemptive antinociceptive effects of EM-1 and EM-2 during 4 h to 4 days (Fig. 3c, *F*_24, 287_ = 23.3, *P* < 0.001; Fig. 4c, *F*_24, 284_ = 23.7, *P* < 0.001, respectively). The selective kappa-opioid receptor antagonist nor-BNI (10 nmol, i.t.) significantly reduced the preemptive anti-allodynia of EM-2 from 4 h to 4 days (Fig. 4d, *F*_24, 269_ = 12.8, *P* < 0.001), but was ineffective in the response to preemptive intrathecal administration of EM-1 (Fig. 3d). As expected, at the same doses, i.t. administration of these opioid antagonists alone had no significant effects in the present pain model.

### The mu-opioid receptor immunoreactivity was increased in DRG neurons after CFA treatment

**Fig. 5:**
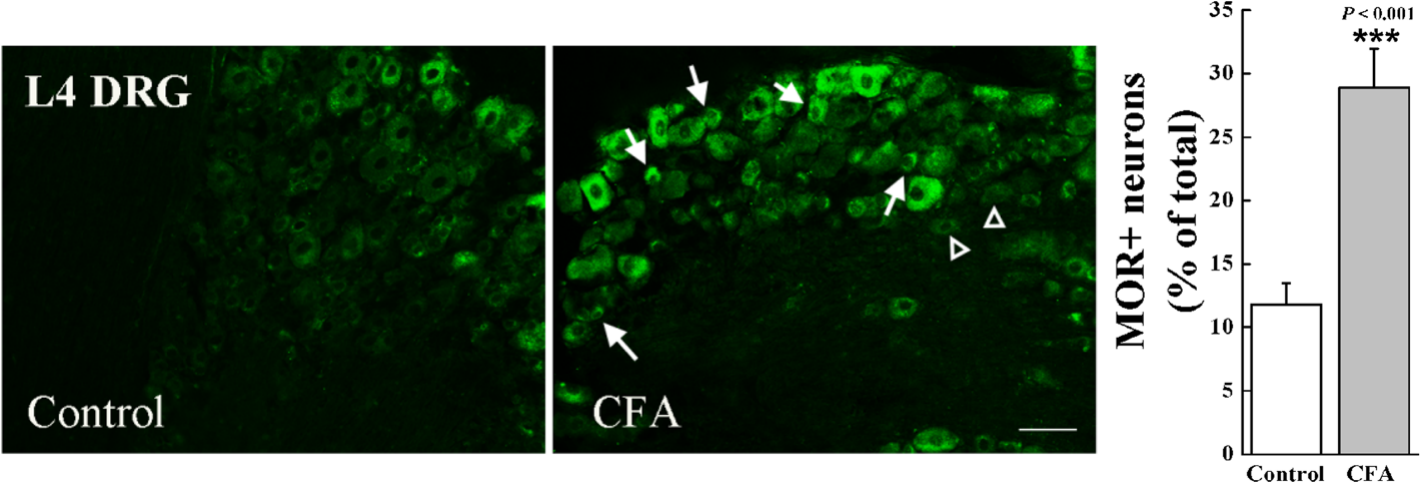
Immunostaining analysis showed that the expression of the mu-opioid receptor is significantly increased after CFA. Photomicrographs of representative L4 DRG neurons illustrated the mu-opioid receptor immunoreactivity neurons (green) in the control group (Control) and CFA group (CFA). Arrows indicate the mu-opioid receptor positive neurons, and triangles indicate the negative neurons. *n* = 4–6 animals/group, ***P < 0.001 indicates significant differences compared with the control group according to two-tailed *t* test. Scale bar = 50 μm

In line with previous studies [41, 42], our immunostaining also showed that the mu-opioid receptor was expressed in DRG neurons (Fig. 5), which is further confirmed with a co-labeling of NeuN (a pan-neuronal marker) (Additional file 2: Figure S1A). More importantly, an increased expression of the mu-opioid receptor in DRG neurons was detected after CFA. In control mice, the percentage of the mu-opioid receptor-positive profiles among all neurons in L4 DRG was 11.76 ± 1.73% (922 positive neurons among a total of 8096 neurons derived from six sections/mouse, *n* = 9 mice). One day after CFA, a significant increase of the mu-opioid receptor-positive neurons (28.88 ± 3.09%, 2857 positive neurons among a total of 8632 neurons derived from six sections/mouse, *n* = 10 mice) was observed (*t*_17_ = − 4.69, *P* < 0.001 relative to control).

### Effects of preemptive intrathecal administration of EM-1 and EM-2 on p38 MAPK in DRG

**Fig. 6:**
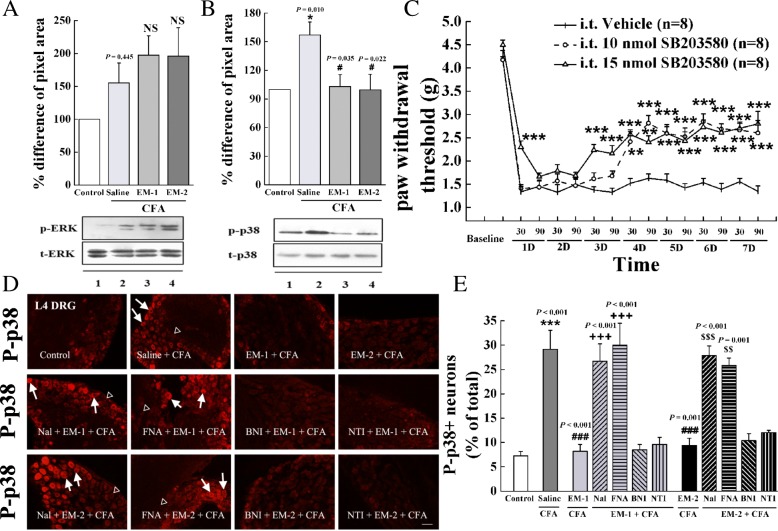
The involvement of p38 MAPK signal in the preemptive analgesia of endomorphins. **a**, **b** Quantitative Western blot analysis of the expression of ERK1/2 and p38 MAPK was performed in ipsilateral DRG tissues. Representative Western blots showed the levels of phosphorylated p38 MAPK or ERK1/2 (top) and total p38 MAPK or ERK1/2 (bottom) in DRG. Lane 1: Control; lane 2: Saline + CFA; lane 3: EM-1 + CFA; lane 4: EM-2 + CFA. Data are presented as the percentage difference relative to the control (*n* = 5, *P < 0.05 vs. Control, ^#^*P <* 0.05 vs. Saline). **c** Repeated administration of the p38 MAPK inhibitor SB203580 (10 and 15 nmol, i.t.) significantly reduced CFA-induced mechanical allodynia (^**^P < 0.01, ***P < 0.001 vs. Vehicle). **d**, **e** Representative photomicrographs of P-p38-immunoreactive neurons (red) in ipsilateral L4 DRG neurons and a graph quantifying the P-p38 expression in the control group, saline-treated CFA group, and endomorphins-treated CFA group were showed. Preemptive intrathecal administration of endomorphins robustly suppressed the increase of P-p38 MAPK immunoreactive neurons as compared with the saline-treated group, and pretreatment with naloxone and β-FNA inhibited the effect of endomorphins. Arrows indicate P-p38 MAPK positive neurons, and triangles indicate the negative neurons (*n* = 4–6 animals/group, scale bar = 50 μm, ***P < 0.001 vs. Control, ^###^*P <* 0.001 vs. Saline, ^++*+*^*P <* 0 .001 vs. EM-1 and ^$$$^*P <* 0.001 vs. EM-2). Data obtained from **a**, **b**, and **e** were statistically analyzed according to one-way ANOVA followed by Bonferroni post-hoc analysis, and data obtained from **c** were statistically analyzed according to two-way ANOVA followed by Bonferroni post-hoc analysis

Previous studies have shown that MAPK pathways, in particular ERK1/2 and p38 MAPK, play an important role in pain hypersensitivity [25]. Thus, to investigate whether MAPK signaling is involved in the preemptive analgesia of endomorphins in inflammatory pain model, we further examined the effects of EM-1 and EM-2 on CFA-induced activation of p38 MAPK and ERK1/2 in DRG neurons. As shown in Fig. 6a, b, Western blot analysis revealed that the phosphorylation of ERK1/2 and p38 MAPK was dramatically increased 1 day after CFA compared with control animals (*F*_3, 19_ = 2.31*, P* = 0.115; *F*_3, 23_ = 5.20*, P* = 0.008, respectively). Notably, preemptive intrathecal administration of EM-1 or EM-2 significantly prevented CFA-induced activation of p38 MAPK (EM-1, *P* = 0.035; EM-2, *P* = 0.022, respectively), but did not modify the upregulation of ERK1/2 phosphorylation.

Consistent with the previous study [42], the co-labeling of P-p38 MAPK and NeuN revealed P-p38 MAPK immunoreactivity in DRG neurons (Additional file 2: Figure S1B). Immunofluorescence staining data further demonstrated that a larger number of DRG neurons exhibited immunoreactivity to phosphorylated p38 MAPK 1 day after CFA (Fig. 6d, *F*_11, 47_ = 17.5*, P* < 0.001; 1205 positive neurons among a total of 4268 neurons derived from six sections/mouse, *n* = 4 mice). Preemptive intrathecal administration of EM-1 or EM-2 significantly decreased CFA-induced upregulation of phosphorylated p38 MAPK in DRG (254 positive neurons among a total of 3302 neurons derived from 4 sections/mouse, *n* = 4 mice, *P* < 0.001; 300 positive neurons among a total of 3156 neurons derived from four sections/mouse, *n* = 4 mice, *P* < 0.001, respectively). In addition, pretreatment with naloxone and β-FNA, but not nor-BNI or NTI, significantly reversed the effect of endomorphins on phosphorylated p38 MAPK signal (for EM-1, naloxone, *P* < 0.001; β-FNA, *P* < 0.001; for EM-2, naloxone, *P* < 0.001; β-FNA, *P* = 0.001).

**Fig. 7:**
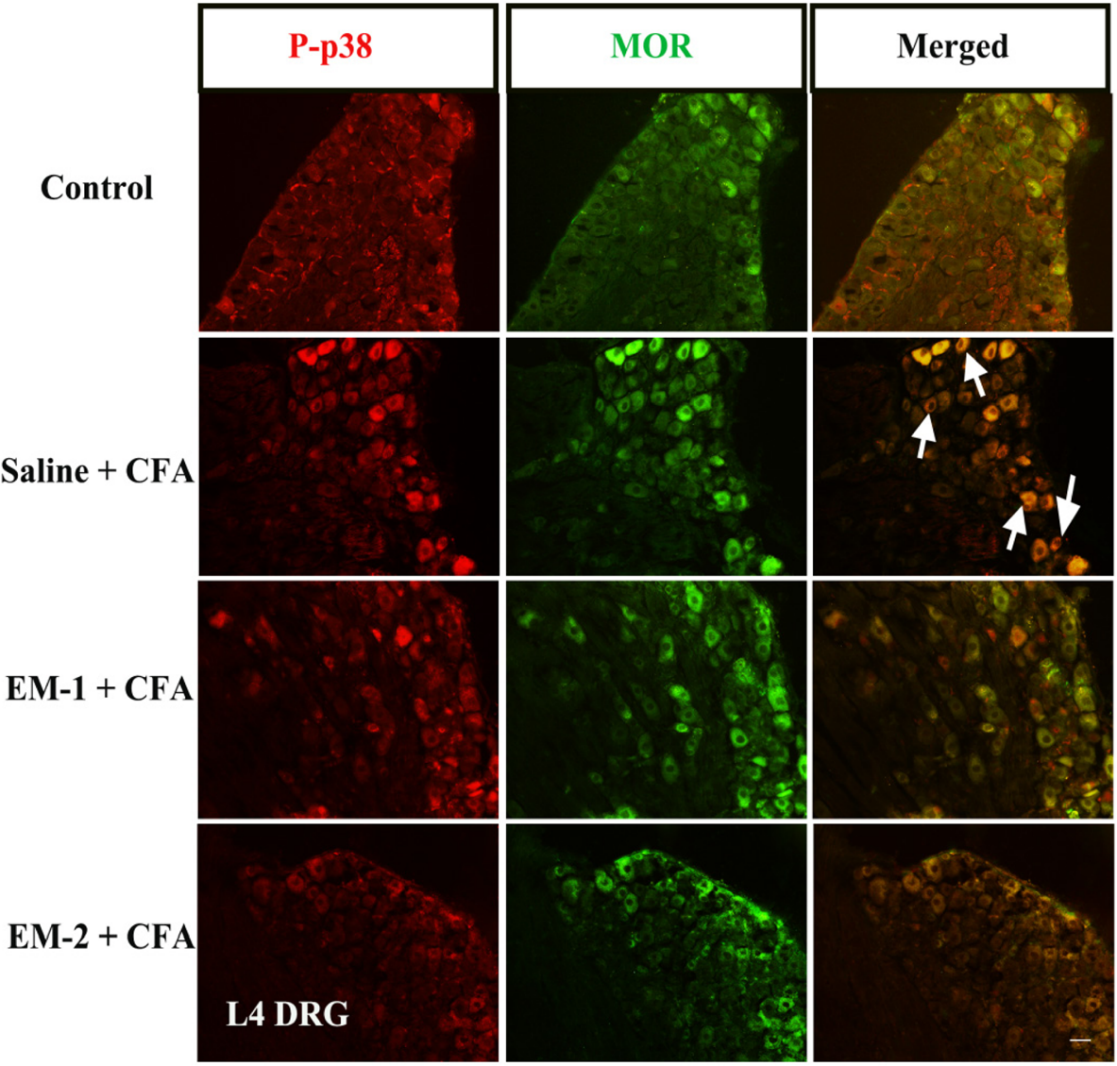
Representative immunofluorescence staining revealed co-expression patterns of P-p38 MAPK with MOR. Double immunostaining of P-p38 MAPK (red) and MOR (green) was investigated in L4 DRG neurons of the control group, saline-treated CFA group, and endomorphins-treated CFA group. Arrows indicate co-expression. *n* = 4–6 animals/group, > 500 neurons per animal were assessed. Scale bar = 50 μm

Next, we performed co-immunolabeling of phosphorylated p38 MAPK with the mu-opioid receptor in DRG neurons from CFA-treated mice. As shown in Fig. 7 and Table 2, the proportion of phosphorylated p38 MAPK and the mu-opioid receptor co-localized neurons was 17.62 ± 0.77% among all DRG neurons in saline-treated CFA mice (*F*_3, 15_ = 40.5*, P* < 0.001, 469 positive neurons among a total of 3367 neurons derived from 6 sections/mouse, *n* = 4 mice), suggesting a significant increase compared with that of control mice (2.62 ± 0.39%, 64 positive neurons among a total of 3570 neurons derived from 6 sections/mouse, *n* = 4 mice). In addition, compared with saline-treated CFA group, preemptive intrathecal administration of EM-1 or EM-2 significantly reduced co-expression of phosphorylated p38 MAPK with the mu-opioid receptor (207 positive neurons among a total of 2814 neurons derived from 6 sections/mouse, n = 4 mice, *P* < 0.001; 209 positive neurons among a total of 2152 neurons derived from 6 sections/mouse, *n* = 4 mice, *P* < 0.001, respectively).

**Table 2:** Expression or co-expression of MOR and P-p38 MAPK in DRG neurons (%: immunoreactive neurons relative to total DRG neurons)

	Control	Saline + CFA	EM-1 + CFA	EM-2 + CFA
P-p38	7.3 ± 0.9	29.2 ± 3.9 ***	8.2 ± 1.4 ^###^	9.4 ± 1.5 ^###^
MOR	11.8 ± 1.7	28.9 ± 3.1 ***	—	—
MOR + P-p38	2.6 ± 0.4	17.6 ± 0.8 ***	7.4 ± 0.9 ^###^	9.8 ± 0.7 ^###^

Quantification illustrates the expression of MOR and P-p38 MAPK and their co-expression in DRG neurons in response to CFA

^***^P < 0*.*001 indicates significant differences compared with Control samples according to one-way ANOVA followed by Bonferroni post-hoc analysis or two-tailed *t* test

^###^*P <* 0*.*001 indicates significant differences compared with Saline + CFA samples according to one-way ANOVA followed by Bonferroni post-hoc analysis

### Intrathecal injection of the p38 MAPK inhibitor SB203580 attenuated CFA-induced mechanical allodynia

To evaluate the functional role of p38 MAPK signaling pathway in CFA-induced inflammatory pain, the selective p38 MAPK inhibitor SB203580 was repeatedly administered once daily for 7 days. Figure 6c showed that repetitive saline-treated mice had the intact development of mechanical allodynia after CFA treatment, while repeated administration of SB203580 (10 and 15 nmol, i.t.) inhibited the activation of p38 MAPK and resulted in a significant anti-allodynic effect (*F*_22, 359_ = 5.72, *P* < 0.001).

### Effects of preemptive intrathecal administration of EM-1 and EM-2 on CFA-induced gene expressions of inflammatory cytokines and chemokines in DRG

**Fig. 8:**
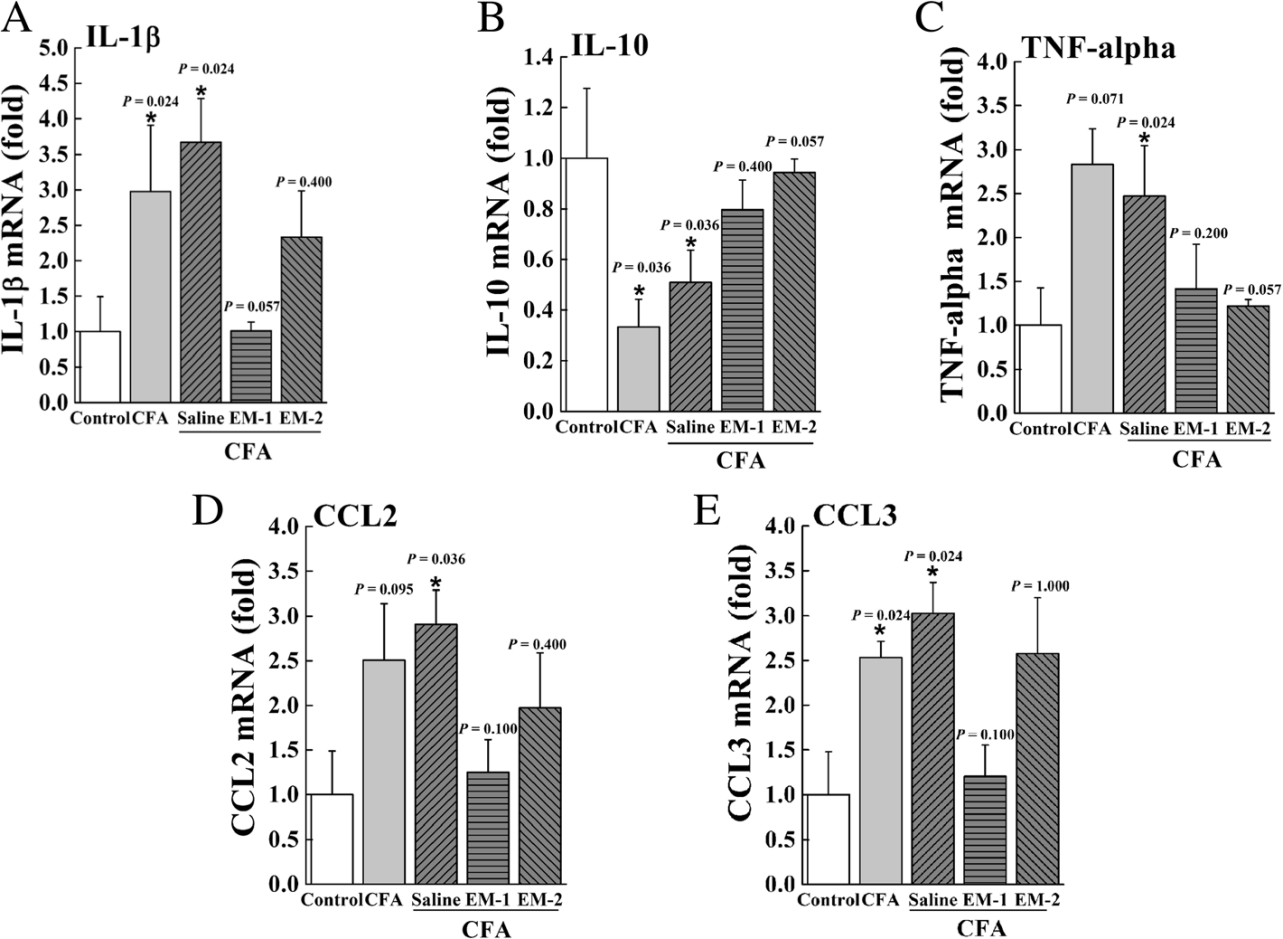
Effects of preemptive intrathecal administration of endomorphins on gene expression of inflammatory cytokines and chemokines. EM-1 and EM-2 differently reversed CFA-induced regulation of mRNA expression of IL-1β **(a)**, IL-10 **(b)**, TNF-alpha **(c)**, CCL2 **(d)**, and CCL3 **(e)** in the ipsilateral DRG tissues 1 day after CFA. Results are shown as the fold expression relative to control (*n* = 8–10 animals/group). *P < 0.05 indicates significant differences compared with Control group according to Mann-Whitney test

Pro-inflammatory cytokines and chemokines were reported to play important roles in the generation and maintenance of chronic pain. To determine the effects of preemptive intrathecal administration of endomorphins on the production of pro-inflammatory mediators, the mRNA levels of IL-1β, TNF-α, CCL2, and CCL3 in ipsilateral DRG tissues were measured by real-time PCR experiments. As shown in Fig. 8 and Table 3, CFA-induced peripheral inflammation substantially increased the expressions of pro-inflammatory mediators, including IL-1β (*P* = 0.024), TNF-α (*P* = 0.024), CCL2 (*P* = 0.036), and CCL3 (*P* = 0.024). Pretreatment with EM-1 produced a slight, but not statistically significant, decrease in CFA-induced upregulation of IL-1β (*P* = 0.057). In addition, preemptive administration of EM-2 substantially reduced the upregulation of TNF-α (*P* = 0.057) induced by CFA. However, neither EM-1 nor EM-2 had significant effects on the expressions of CCL2 (EM-1, *P* = 0.100; EM-2, *P* = 0.400) and CCL3 (EM-1, *P* = 0.100; EM-2, *P* = 1.000) in inflammatory animals.

**Table 3:** Relative mRNA levels of inflammatory mediators in DRG tissues

Gene	CFA	Saline + CFA	EM-1 + CFA	EM-2 + CFA
IL-1β	2.46 ± 0.70	3.67 ± 0.61*	1.08 ± 0.16 ^#^	2.33 ± 0.57
IL-10	0.33 ± 0.11**	0.51 ± 0.13*	0.80 ± 0.12	0.77 ± 0.23
TNF-α	2.84 ± 0.41*	2.07 ± 0.60	1.41 ± 0.51	1.21 ± 0.08
CCL2	2.51 ± 0.63	2.91 ± 0.38*	1.25 ± 0.37	1.97 ± 0.61
CCL3	2.20 ± 0.44**	3.03 ± 0.34**	1.21 ± 0.35	2.58 ± 0.63

Inflammatory mediators mRNA levels are assessed by RT-PCR assay and normalized to GAPDH-expression

^*^P < 0*.*05 and ^**^P < 0*.*01 indicates significant differences compared with Control samples according to one-way ANOVA followed by Bonferroni post-hoc analysis

^#^*P <* 0*.*05 indicates significant differences compared with Saline + CFA samples according to one-way ANOVA followed by Bonferroni post-hoc analysis

IL-10 is an anti-inflammatory cytokine and has a crucial role in the prevention of inflammatory diseases since it reduces the production of pro-inflammation mediators [43]. In Fig. 8b, our results indicated that the mRNA level of IL-10 in ipsilateral DRG 1 day after CFA treatment was significantly lower than that of control animals (*P* = 0.036). Moreover, pretreatment with EM-2 produced a slight, but not statistically significant, increase in CFA-induced downregulation of IL-10 expression (*P* = 0.057).

## Discussion

Endomorphins were characterized as the selective mu-opioid agonists and held great promise in the design of new candidate drugs [18, 44]. Recently, several series of endomorphin analogs with potent and long-lasting antinociception provide lead compounds for the development of novel analgesics [7, 44]. Our results demonstrated that preemptive intrathecal administration of endomorphins produced potent anti-allodynic effects in CFA-induced inflammatory pain model. As a result of inflammation, mice exhibited a significant activation of p38 MAPK pathway and an increased expression of inflammatory cytokines in DRG tissues, which were partially reconciled by preemptive administration of endomorphins. In conclusion, our data implicate that preemptive intrathecal administration of endomorphins reduces inflammatory pain via inhibition of p38 MAPK phosphorylation and regulation levels of inflammatory cytokines in DRG.

Endomorphins and their analogs were reported to produce potent analgesic activities in acute and chronic pain models [2–4, 7]. However, the preemptive analgesic effects of endomorphins on chronic pain were poorly uncovered. Here, our research for the first time showed that in a mouse model of CFA inflammatory pain, endomorphins dose-dependently produced preemptive analgesic effects after i.t. administration in a manner similar to the endogenous opioid ligand nociceptin/orphanin FQ (N/OFQ) [17, 45]. Of note, central administration of endomorphins produced acute antinociceptive actions which only sustained for 20–30 min [13, 14]. Mizoguchi et al. reported that i.t. administration of EM-1 and EM-2 effectively inhibited the paw-withdrawal responses within 30 min [13]. In striking contrast, we found that preemptive intrathecal administration of EM-1 and EM-2 relieved CFA-induced inflammatory pain for 6 days. Moreover, endomorphins also produced preemptive anti-allodynia for 2 days in postoperative pain model. Our findings suggest the prolonged anti-allodynic effects produced by preemptive antinociception of endomorphins might be due to their abilities of blocking the development of pain. In fact, preemptive treatment with analgesics prevents or blunts spinal facilitation evoked by nociceptive input to the spinal cord and attenuates perceived pain in a long-lasting manner [42, 45, 46]. The preemptive administration of analgesics might be an attractive strategy for the treatment of chronic pain.

Furthermore, the preemptive analgesic effects of EM-1 and EM-2 were completely blocked by the opioid receptor antagonist naloxone, suggesting the involvement of opioid system. Our present data indicated the significant antagonism with the selective mu-opioid receptor antagonist β-FNA, which further implies that both EM-1 and EM-2 produce preemptive analgesic effects via the mu-opioid receptor. These findings are consistent with the previous results that both EM-1 and EM-2 activated the mu-opioid receptor with high affinity and selectivity [1]. Previous research found that the endogenous delta-opioid ligand met-enkephalin was expressed in inflamed subcutaneous tissue [47]. Our results showed that EM-1- and EM-2-induced preemptive analgesia were partially reduced by the selective delta-opioid receptor antagonist NTI. However, i.t. administration of the selective kappa-opioid receptor antagonist nor-BNI partially attenuated the preemptive analgesic effect of EM-2, but not that of EM-1. The different pharmacological profiles of EM-1 and EM-2 in this study are in line with the previous report that spinal antinociception of EM-1 and EM-2 is mediated by different subtypes of the mu-opioid receptor [48]. Spinal antinociception of EM-2 is mediated through activation of the mu(1)-opioid receptor which subsequently induced the release of the endogenous kappa-opioid peptide dynorphin A-(1-17) in the spinal cord [48]. Based on these results, we speculate that endomorphins-induced preemptive analgesia might be partially mediated by release of endogenous opioid peptides.

Previous studies have shown that the expression of the mu-opioid receptor mRNA is upregulated in DRG tissues by painful peripheral inflammation [49]. Here, we extended this study and found that the mu-opioid receptor at the protein level was significantly increased in ipsilateral DRG neurons 1 day after CFA injection. Considering the antinociceptive effects of endomorphins in the present study, CFA-induced activation of the mu-opioid receptor system in DRG may be associated with the suppression of nociceptive hypersensitivity in CFA-induced inflammatory pain model.

It is well known that MAPK pathways, especially ERK1/2 and p38 MAPK, modify neuronal plasticity and contribute to pain hypersensitivity [25]. A remarkable increase of phosphorylated p38 MAPK and ERK1/2 was detected in spinal cord and DRG tissues in acute and persistent inflammatory pain models [26, 50–52]. In addition, intrathecal injection of the selective inhibitors of MAPK pathways produced analgesic activities in rodent models of inflammatory, neuropathic and cancer pain [25]. The present study showed that the levels of phosphorylated ERK1/2 and p38 MAPK in DRG tissues were increased 1 day after CFA treatment, corroborating the previous findings [26, 52]. In addition, pretreatment with endomorphins selectively attenuated CFA-induced phosphorylation of p38 MAPK, which was reversed by pretreatment with the mu-opioid receptor antagonist. Furthermore, our results demonstrated that repeated i.t. injection of the p38 MAPK inhibitor SB203580 partially prevented the development of mechanical hypersensitivity induced by CFA. Taken together, our current data suggest the preemptive analgesic effects of endomorphins might be mediated by suppression of p38 MAPK pathway, but independent of ERK1/2.

To date, several studies have reported a functional interaction between the activation of the mu-opioid receptor and the phosphorylation of MAPK signaling in both in vivo and in vitro assays. For example, fentanyl selectively activated ERK1/2 signal, but not p38 MAPK signal in striatal neurons [53]. In vitro study found that EM-1 could elicit rapid activation of ERK1/2 in rat C6 glioma cells [54], and morphine induced a decrease of IL-10 and an increase of IL-12 secretion via p38 MAPK pathway in monocyte-derived human dendritic cells [55]. Furthermore, Li et al. found that endomorphins decreased the activation of p38 MAPK induced by LPS, but not that of ERK1/2 in murine dendritic cells [30]. Therefore, it is possible that the mu-opioid system might have a differential modulation on the activity of p38 MAPK or ERK1/2, depending on cell types, treatments, or in vitro or in vivo studies, etc.

Neuroinflammation is crucial pathogenesis of chronic pain [56, 57]. Numerous studies provided evidence that pro-inflammatory cytokines, such as TNF-α, IL-1β, and IL-6, were upregulated in the spinal cord after nerve injury, inflammation, and chronic opioid exposure, which contribute in inducing or facilitating inflammatory responses as well as mechanical allodynia [58]. Both TNF-α and IL-1β played essential roles in the generation of central sensitization and modulation of peripheral sensitization [59, 60]. The inhibition of IL-1β signaling was reported to block the development of inflammatory, neuropathic, and cancer pain [61]. Here, we found that intraplantar injection of CFA significantly upregulated the mRNA expressions of IL-1β and TNF-α on day 1 post-injection, which is consistent with the previous report [58]. Importantly, we found that the upregulation of IL-1β was substantially reduced by EM-1, while the activation of TNF-α was regulated by EM-2. IL-10 is an anti-inflammatory cytokine which suppresses the production of pro-inflammatory cytokines and the induction of hyperalgesia and allodynia in different pain models [43]. Although it did not achieve a significant difference, EM-2 substantially reduced the downregulation of IL-10 induced by CFA. Thus, the regulation of IL-1β, TNF-α, and IL-10 levels might be involved in EM-1- and EM-2-induced preemptive anti-allodynia, respectively. At present, there is no evidence to document the differential effects of EM-1 and EM-2 on the expressions of inflammatory cytokines. Of note, previous studies found that spinal antinociception of EM-1 and EM-2 are mediated by different mu-opioid receptor subtypes [13, 48]. Whether this could account for the differential effects of EM-1 and EM-2 on the expression of inflammatory cytokines needs further studies.

Chemokines can regulate chronic pain by modulating the signaling interactions between neurons and glial cells [62]. Previous studies have shown that nerve injury and inflammation upregulated the expressions of CCL2, CCL3, and CCL21 in DRG tissues [63, 64]. A great number of studies found that the mu-opioid receptor and chemokine receptors, such as CCR5, CCR1, CXCR4, and CX3CR1, are co-expressed on the same population of DRG neurons [65]. Moreover, chemokines also participated in the analgesic effects of opioids [65]. Our results demonstrated that CCL2 and CCL3 mRNAs were dramatically increased 1 day after CFA. Interestingly, we found that EM-1 and EM-2 had no significant effects on the gene expressions of CCL2 and CCL3 in the present study.

In addition, accumulating evidence has indicated that various inflammatory cytokines and chemokines produced by DRG satellite glial cells and released to the extracellular space contribute to the development and maintenance of chronic pain [59]. Our results showed that intrathecal pretreatment with endomorphins inhibited the activation of satellite glial cells in DRG tissues (Additional file 3: Figure S2), suggesting a possible involvement of non-neuronal mechanism in analgesia of preemptive treatment of endomorphins. Further mechanistic studies are under investigation in our lab.

## Conclusions

In summary, our research provides, for the first time, preemptive analgesic effects of spinal endomorphins in CFA-induced inflammatory pain. Several lines of evidence suggest that the preemptive analgesic effects of endomorphins are associated with the regulation of inflammatory mediators via inhibiting p38 MAPK signal in DRGs. In brief, (1) peripheral inflammation increases the expression of phosphorylated p38 MAPK, which is attenuated by preemptive intrathecal administration of endomorphins, (2) pretreatment with the opioid receptor antagonists naloxone and β-FNA significantly reverses endomorphins-induced effects on phosphorylated p38 MAPK signal in DRG, (3) repeated application of the p38 MAPK inhibitor SB203580 significantly reduces CFA-induced mechanical allodynia, (4) co-localization of phosphorylated p38 MAPK with the mu-opioid receptor in DRG neurons is increased after inflammation, which is attenuated by preemptive intrathecal administration of endomorphins. These findings support the involvement of p38 MAPK signal in the preemptive analgesia of endomorphins. Moreover, preemptive intrathecal administration of endomorphins regulates the expressions of cytokines in ipsilateral DRG tissues. Therefore, our study indicates that the preemptive analgesia of endomorphins may be helpful for chronic pain treatment.

## Additional files

Additional file 1: Supplemental methods. Table S1 Primary and secondary antibody information. (DOCX 25 kb)

Additional file 2: **Figure S1.** Phosphorylated p38 MAPK and the mu-opioid receptor were expressed in mouse ipsilateral L4 DRG neurons. (**A**) Double immunostaining of MOR (green) and NeuN (red) was investigated in L4 DRG tissues of control group and saline-treated CFA group. (**B**) Double immunostaining of P-p38 MAPK (red) and NeuN (green) was conducted in L4 DRG tissues of control group and saline-treated CFA group. Arrows indicate co-expression. *n* = 4 animals/group, > 500 neurons per animal were assessed. Scale bar = 50 μm. (PPTX 2054 kb)

Additional file 3: **Figure S2.** Preemptive administration of EM-1 and EM-2 inhibited the activation of glial cells induced by CFA. Representative photomicrographs of the immunoreactivity of GFAP (a marker of satellite glial cells) in ipsilateral L4 DRG and a graph quantifying the expression of GFAP was showed. Immunostaining analysis indicated that the expression of GFAP was significantly increased 1 day after CFA treatment. Preemptive intrathecal administration of endomorphins robustly suppressed the immunoreactivity of GFAP in ipsilateral L4 DRG tissues as compared with the saline-treated group. *n* = 4–6 animals/group, one-way ANOVA followed by Bonferroni post-hoc analysis was used. Scale bar = 50 μm. (PPTX 1192 kb)

## Abbreviations

ANOVA: Analysis of variance
AUC: Area under the curve
ED_50_: Effective dose 50% of maximum response
EM-1: Endomorphin-1
EM-2: Endomorphin-2
i.t.: Intrathecal
IL-1β: Interleukin-1beta
MOR: Mu-opioid receptor
nor-BNI: Nor-binaltorphimine
NTI: Naltrindole
S.E.M.: Standard error of the mean
TNF-α: Tumor necrosis factor-α
β-FNA: Beta-funaltrexamine
