# Supplementary material for: Preemptive intrathecal administration of endomorphins relieves inflammatory pain in male mice via inhibition of p38 MAPK signaling and regulation of inflammatory cytokines
Source: J Neuroinflammation. 2018 Nov 15;15:320. doi: 10.1186/s12974-018-1358-3 (PMC6236886; doi:10.1186/s12974-018-1358-3)
Supplement: Supplementary file 1 — Supplemental methods. Table S1 Primary and secondary antibody information. (DOCX 25 kb) [file 12974_2018_1358_MOESM1_ESM.docx]

**Supplemental Information**

**Supplemental Methods**

***Immunofluorescence staining***

Immunofluorescence staining of GFAP or double immunostaining of NeuN & phosphorylated p38 MAPK and NeuN & the mu-opioid receptor was performed on 12-µm thickness sections of ipsilateral L4 DRG. The sections were blocked in 5% normal donkey serum for 45 min and then incubated with primary antibody (goat polyclonal GFAP, mouse monoclonal NeuN, rabbit monoclonal P-p38 MAPK, guinea pig monoclonal mu-opioid receptor) overnight (4 °C). Detection was performed using an appropriate fluorescent secondary antibody (Alexa Fluor 488 or Alexa Fluor 594). Images were obtained at ×20 magnification under an Olympus fluorescence microscope.

For GFAP labeling, data were measured followed as previously described [1]. Sections were manually outlined using the freehand tool, the threshold was set just above the level of background within the outlined region, and the labeling density was measured using the integrated density algorithm of Image J. Data were analyzed using ANOVA followed by Bonferroni post-hoc analysis. Significance was set at *P* < 0.05. For double label co-localization, images were made into figures using Adobe Photoshop (Adobe Systems Incorporated, USA). Quantification of the percentage of co-localized neurons was determined by counting the number of co-expression neurons and the total numbers of DRG neurons.

**Supplemental Reference**

1. Chen Y, Willcockson HH, Valtschanoff JG. Influence of the vanilloid receptor TRPV1 on the activation of spinal cord glia in mouse models of pain. Exp Neurol. 2009; 220:383-390.

**Supplemental Table**

| Primary antibody | Dilution | Supplier | Cat# | Secondary antibody |
| --- | --- | --- | --- | --- |
| Rb anti-P-p38 MAPK | 1:100 | CST | 4511 | AlexaFluor 594-conjugated GA anti-Rb (1:1000) |
| GP anti-MOR | 1:100 | Neuromics | GP10106 | AlexaFluor 488-conjugated GA anti-GP (1:1000) |
| Ms anti-c-Fos | 1:100 | SCB | sc-8047 | AlexaFluor 488-conjugated GA anti-Ms (1:1000)  AlexaFluor 594-conjugated Dk anti-Ms (1:1000) |
| GA anti-GFAP | 1:500 | SCB | sc-6170 | AlexaFluor 488-conjugated Dk anti-GA (1:1000) |
| Ms anti-NeuN | 1:500 | Abcam | ab104224 | AlexaFluor 488-conjugated GA anti-Ms (1:1000)  AlexaFluor 594-conjugated Dk anti-Ms (1:1000) |

**Table S1** **Primary and secondary antibody information.**

^1^ GA, goat; Ms, mouse; Rb, rabbit; Dk, Donkey; GP, Guinea pig; MOR, mu-opioid receptor.

^2^ SCB: Santa Cruz Biotechnology; CST: Cell signaling Technology, Inc.

^3^ AlexaFluor 594-conjugated Dk anti-Ms antibody purchases from Abcam and all other secondary antibodies are from Life Technologies.
