# Supplementary figures and images for: Preemptive intrathecal administration of endomorphins relieves inflammatory pain in male mice via inhibition of p38 MAPK signaling and regulation of inflammatory cytokines
Source: J Neuroinflammation. 2018 Nov 15;15:320. doi: 10.1186/s12974-018-1358-3 (PMC6236886; doi:10.1186/s12974-018-1358-3)

## Slide 1
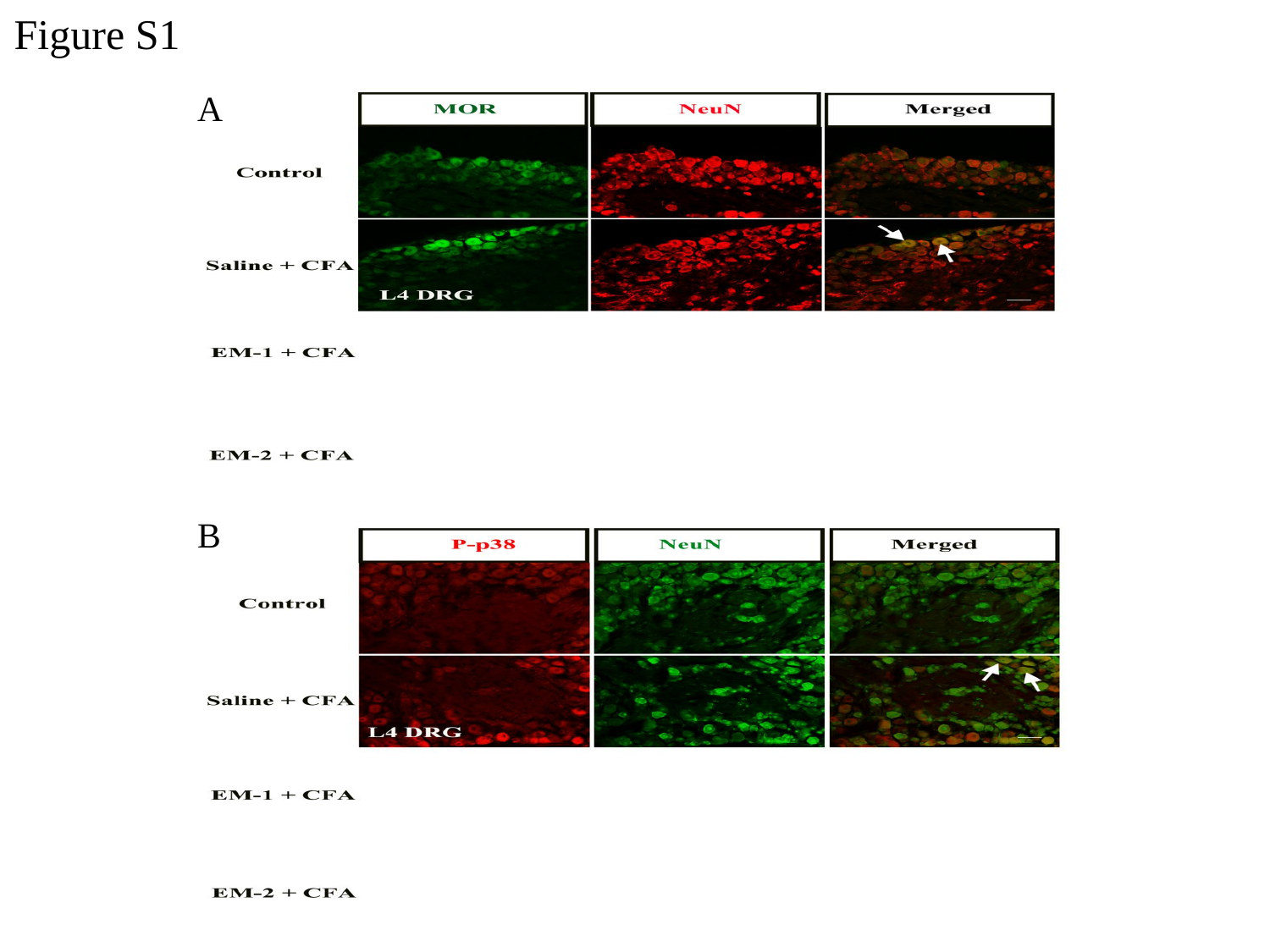

Figure S1
A
B

Supplement: Supplementary file 2 — Figure S1. Phosphorylated p38 MAPK and the mu-opioid receptor were expressed in mouse ipsilateral L4 DRG neurons. (A) Double immunostaining of MOR (green) and NeuN (red) was investigated in L4 DRG tissues of control group and saline-treated CFA group. (B) Double immunostaining of P-p38 MAPK (red) and NeuN (green) was conducted in L4 DRG tissues of control group and saline-treated CFA group. Arrows indicate co-expression. n = 4 animals/group, > 500 neurons per animal were assessed. Scale bar = 50 μm. (PPTX 2054 kb) [file 12974_2018_1358_MOESM2_ESM.pptx]

## Slide 1
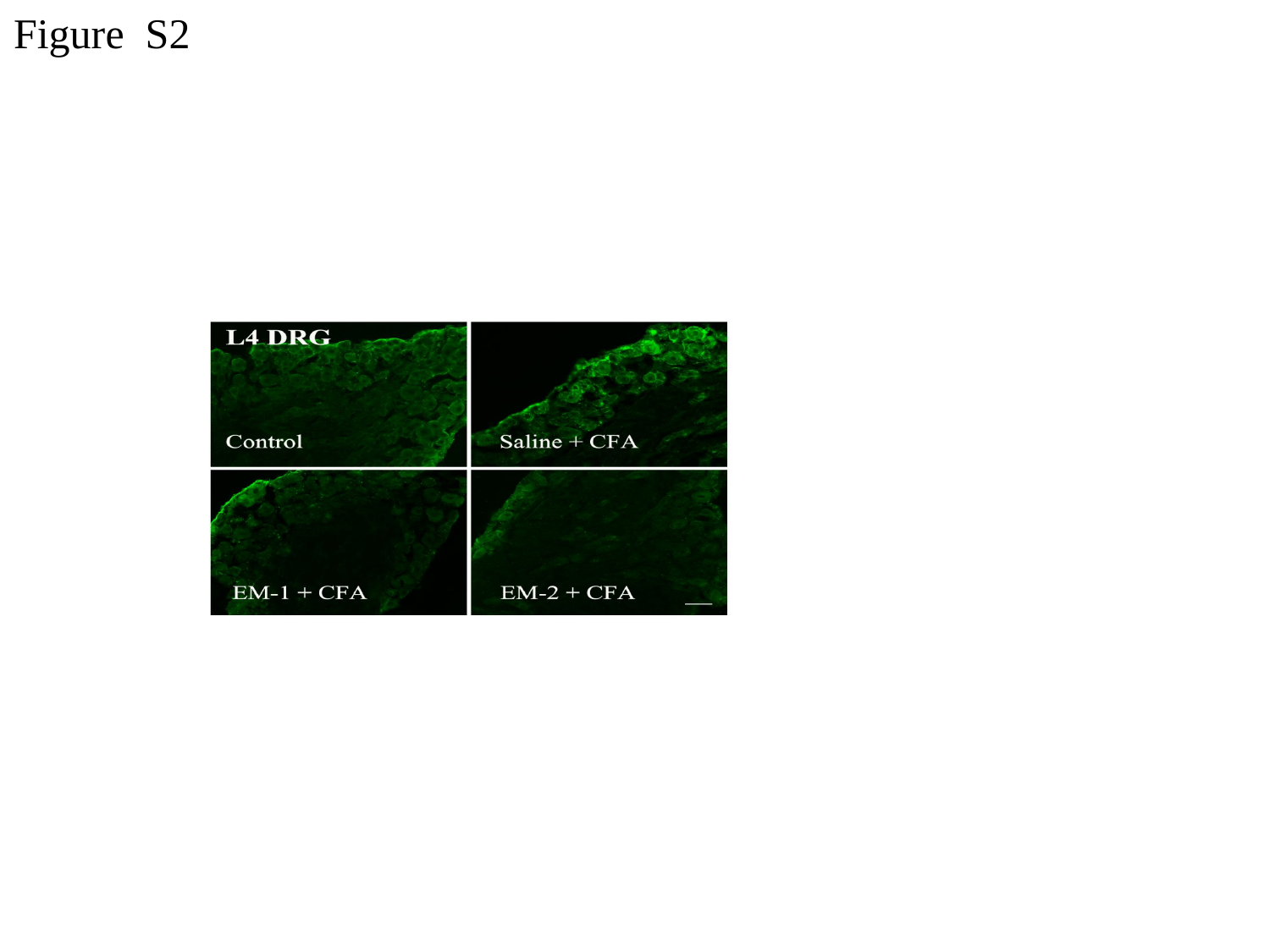

Figure S2

Supplement: Supplementary file 3 — Figure S2. Preemptive administration of EM-1 and EM-2 inhibited the activation of glial cells induced by CFA. Representative photomicrographs of the immunoreactivity of GFAP (a marker of satellite glial cells) in ipsilateral L4 DRG and a graph quantifying the expression of GFAP was showed. Immunostaining analysis indicated that the expression of GFAP was significantly increased 1 day after CFA treatment. Preemptive intrathecal administration of endomorphins robustly suppressed the immunoreactivity of GFAP in ipsilateral L4 DRG tissues as compared with the saline-treated group. n = 4–6 animals/group, one-way ANOVA followed by Bonferroni post-hoc analysis was used. Scale bar = 50 μm. (PPTX 1192 kb) [file 12974_2018_1358_MOESM3_ESM.pptx]
